# Premorbid frailty, stress hyperglycemia ratio, and functional outcome in patients with acute ischemic stroke

**DOI:** 10.3389/fneur.2024.1463814

**Published:** 2024-10-24

**Authors:** Marialuisa Zedde, Simona Lattanzi, Andrea Pilotto, Daniel Janitschke, Jakob Stögbauer, Fatma Merzou, Rosario Pascarella, Alessandro Padovani, Andrea Morotti, Piergiorgio Lochner

**Affiliations:** ^1^Neurology Unit, Stroke Unit, Azienda Unita Sanitaria Locale IRCCS di Reggio Emilia, Reggio Emilia, Italy; ^2^Neurological Clinic, Department of Experimental and Clinical Medicine, Marche Polytechnic University, Ancona, Italy; ^3^Department of Clinical and Experimental Sciences, University of Brescia, Brescia, Italy; ^4^Laboratory of Digital Neurology and Biosensors, University of Brescia, Brescia, Italy; ^5^Neurobiorepository and Laboratory of Advanced Biological Markers, University of Brescia and Azienda Socio Sanitaria Territoriale Spedali Civili Hospital, Brescia, Italy; ^6^Neurology Unit, Department of Continuity of Care and Frailty, Azienda Socio Sanitaria Territoriale Spedali Civili Brescia Hospital, Brescia, Italy; ^7^Department of Neurology, University of the Saarland, Homburg, Saar, Germany; ^8^Neuroradiology Unit, Azienda Unita Sanitaria Locale IRCCS di Reggio Emilia, Reggio Emilia, Italy; ^9^Brain Health Center, University of Brescia, Brescia, Italy

**Keywords:** SHR, frailty, stroke, IVT, MPI

## Abstract

**Background:**

Frailty, defined as multidimensional prognostic index (MPI), has been recently identified as strong predictor of disability and mortality in the elderly with acute ischemic stroke (AIS). The stress hyperglycemia ratio (SHR) is a recently introduced biomarker significantly associated with poor outcome in AIS.

**Objectives:**

This study aimed to investigate in what extent frailty, measured by MPI, and SHR affects the 3-months outcome of patients > 65 years-old with AIS.

**Methods:**

Consecutive patients with AIS >65 years-old who underwent intravenous thrombolysis (IVT) from 2015 to 2019 were enrolled in a German and an Italian Stroke Unit. The SHR was calculated by dividing the fasting plasma glucose at admission with glycated hemoglobin. Demographics and clinical premorbid data, stroke-related variables, including baseline and post-treatment NIHSS score were included in a logistic regression model. The 3-months functional outcome was evaluated by using modified Rankin scale (mRS); good outcome was defined as mRS 0–2, poor as mRS ≥ 3.

**Results:**

One hundred and fifty-five AIS patients were enrolled in the study. Median MPI was 0.19 [0.13–0.31]; 118 (76.1%) patients were classified as “robust” and 37 (23.9%) as “frail.” In regression analysis, age, NIHSS, and MPI demonstrated as the most significant predictor of 3-months good outcome in the whole cohort. In robust patients, SHR values were significantly associated with the outcome.

**Conclusions:**

MPI is associated with the 3-months outcome in our cohort, in particular with good outcome. Conversely, SHR seems to be associated with a 3-months poor outcome in “robust” patients but not in frail patients.

## Introduction

Recent studies demonstrated that premorbid frailty assessed with different measures including the multidimensional frailty index (MPI) is a major determinant of the short and long-term response to acute reperfusion treatment in elderly patients with cerebrovascular events (1, 2). Stress hyperglycemia ratio (SHR) is a different parameter, proposed as a marker of increased risk of short-term mortality and poor functional outcome after ischemic stroke (3, 4).

Some studies have observed that SHR is associated with greater activation of the hypothalamic pituitary axis with an increase of pro-inflammatory cytokines. Another role of SHR is the possible induction of prothrombotic shift and enhance of platelet-endothelial adhesion. Altogether, this can increase induction of endothelial apoptosis, greater inflammation and oxidative stress.

However, the relation between SHR and stroke severity has been demonstrated in young patients and in this category, frailty is not contemplated (5).

In older patients the severity of stroke could be predicted by both frailty and SHR and the independent role of each to another in this issue has not been documented until now. In addition, the majority of studies are about Asian populations and this issue limits the extensions of the findings without further larger studies in other countries (6–8).

In a recent meta-analysis (9), higher SHR significantly increased the occurrence of poor outcomes, mortality, neurological deficit, haemorrhagic transformation, and infectious complications independent of the presence of diabetes and the type of reperfusion treatment. MPI and SHR might represent independent predictors of outcome in stroke but their performance has never been assessed in the same cohort to the best of our knowledge.

Therefore, in the present study, we investigated the role of both indexes, MPI and SHR, as predictors of poor outcome in AIS patients treated with intravenous thrombolysis (IVT).

## Materials and methods

### Study participants

The required sample size had not been calculated beforehand because we performed an exploratory analysis of the already available data of the Italian and German cohorts and were planning to extend this exploration based on the findings.

Data from all patients with acute ischemic stroke (AIS), consecutively admitted to Stroke Units in the Department of Neurology, Homburg, Germany and Ancona, Italy between Jan 1 2015 and December 31, 2019 and treated by IVT, were collected. Inclusion criteria were: (1) age > 65 years; (2) diagnosis of AIS confirmed by brain imaging; (3) IVT with rtPA with or without endovascular thrombectomy (EVT); (4) informed consent for the use of clinical data and 3-months follow-up; (5) the availability of information for the calculation of premorbid MPI. The Institutional Ethical Standards Committee on human experimentation at Saarland Hospital provided approval for the study (ID 269/17), same did the Ethics Committee of Marche Polytechnic University (ID 57/2020).

### Frailty assessment

In order to calculate the MPI, a comprehensive geriatric assessment (CGA) including eight domains (comorbidity index, number of drugs, pressure scores, dependency on basic and instrumental activities of daily living, cognitive, nutritional and social status) was applied (6). The sum of scores of each domain was divided by eight to obtain a final MPI risk score between 0 = no risk and 1 = higher risk of mortality (Supplementary material). The patients were thus dichotomized as robust or frail (MPI value < and > 0.34, respectively) (10).

### Assessment of stress hyperglycemia

Fasting plasma glucose levels were monitored shortly after admission before IVT. Glycosylated hemoglobin (HbAlc) was measured within 24 h after hospitalization, and stress hyperglycemia ratio was calculated as fasting glucose (mmol/L)/HbA1c (%).

### Study outcome

The 3-months modified Rankin Scale (mRS) score, derived by two trained physicians through telephonic interviews, was used to evaluate the functional outcome. Poor outcome was defined as mRS ≥ 3.

### Statistical analysis

Continuous variables were summarized as median [interquartile range (IQR)], and categorical variables were presented as the number (%) of patients. The Mann-Whitney test or chi-squared test were used for univariate comparisons. Logistic regression was used to explore the relationship between the SHR and poor 3-month outcome in the overall study cohort and according to the frailty status (i.e., frail and robust patients, as defined according to the MPI values). Age, baseline NIHSS, and endovascular treatment were selected for the multivariate model as deemed strong potential confounders; the limited sample size did not allow to include additional variables and prevented to perform subgroup analyses according to the status of diabetes mellitus. Results were reported as odds ratio (OR) with associated 95% confidence interval (CI). Statistical significance was set at *p*-values < 0.05. STATA/IC 13.1 statistical package (StataCorp LP, College Station, TX, USA) was used to perform statistical analysis.

## Results

A total amount of 155 patients with AIS were included in this study (Supplementary Figure 1). The median age of the participants was 77 [71–81] years and 80 (51.6%) were men. The median NIHSS score on admission was 12 [6–16] and the median SHR was 1.20 [1.04–1.36]. IVT was performed in all patients and EVT was associated in 90 (58.1%) cases.

The median MPI in the study cohort was 0.19 [0.13–0.31]; 118/155 (76.1%) patients were classified as “robust” and 37/155 (23.9%) as “frail.” The clinical features of patients according to their frailty status are summarized in Table 1. Frail patients were older, had higher SHR values but similar cardiovascular risk factors.

**Table 1:** Baseline characteristics of study participants according to frail status.

	**Full cohort (*n* = 155)**	**Robust (*n* = 118)**	**Frail (*n* = 37)**	***p*-value**
Age, yr	77 [71–81]	75 [71–79]	83 [79–84]	< 0.001^a^
Male sex	80 (51.6)	64 (41.3)	16 (10.3)	0.243^b^
Diabetes mellitus	47 (30.3)	35 (29.7)	12 (32.4)	0.749^b^
Hypertension	122 (78.7)	90 (76.3)	32 (86.5)	0.185^b^
Atrial fibrillation	50 (32.3)	34 (28.8)	16 (43.2)	0.101^b^
Dyslipidemia	56 (36.1)	38 (32.2)	18 (48.7)	0.069^b^
Coronary heart disease	25 (16.1)	18 (15.3)	7 (18.9)	0.597^b^
Smoking	27 (17.4)	22 (18.6)	5 (13.5)	0.473^b^
MPI	0.19 [0.13–0.31]	0.16 [0.06–0.25]	0.44 [0.38–0.56]	< 0.001^a^
Baseline NIHSS	12 [6–16]	12 [6–16]	14 [8–17]	0.281^a^
Anterior circulation territory	106 (68.4)	76 (64.4)	30 (81.8)	0.057^b^
Endovascular treatment	90 (58.1)	69 (58.5)	21 (56.8)	0.853^b^
Stress hyperglycemia ratio	1.20 [1.04–1.36]	1.18 [1.03–1.35]	1.28 [1.15–1.48]	0.020^a^
Poor 3-month outcome^#^	94 (60.6)	63 (53.4)	31 (83.8)	< 0.001^b^

Data are presented as median [IQR] for continuous variables, and n (%) for categorical variables.

^a^Mann-Whitney test.

^b^Chi-squared test.

^#^Poor outcome was defined as mRS ≥ 3.

Table 2 shows baseline characteristics of the study participants according to SHR. Poor 3-month outcome occurred in 94/155 (60.7%) participants of the study cohort, and it was more common among frail compared to robust patients (83.8 vs. 53.4%; *p* < 0.001). In the study cohort, higher NIHSS values at baseline (OR 1.16, 95% CI 1.07–1.27 for unitary increase of NIHSS; *p* < 0.001), increased SHR (OR 8.10, 95% CI 1.68–39.09 for unitary increase of SHR; *p* = 0.009), and higher MPI values (OR 80.09, 95% CI 3.90–1,644.324 for unitary increase of MPI; *p* = 0.004), were independently associated with increased risk of poor functional outcome at 3 months (Table 3). Among robust patients, older age (OR 1.09, 95% CI 1.01–1.17 for unitary increase of age; *p* = 0.022), higher NIHSS values at baseline (OR 1.13, 95% CI 1.03–1.24 for unitary increase of NIHSS; *p* = 0.008), and increased SHR (OR 8.62, 95% CI 1.61–46.12 for unitary increase of SHR; *p* = 0.012), were independently associated with increased odds of poor 3-month functional outcome (Table 4). Among frail patients, the baseline NIHSS (OR 1.29, 95% CI 1.03–1.63; *p* = 0.028) was the only predictor of 3-month functional status, being higher values associated with an increased risk of poor outcome (Table 5).

**Table 2:** Baseline characteristics of study participants according to stress hyperglycemia ratio.

	**Low SHR (*n* = 78)**	**High SHR (*n* = 77)**	***p*-value**
Age, yr	76 [71–80]	78 [73–82]	0.125^a^
Diabetes mellitus	17 (21.8)	30 (39.0)	0.020^b^
Hypertension	59 (75.6)	63 (81.8)	0.348^b^
Atrial fibrillation	25 (32.1)	25 (32.5)	0.956^b^
Dyslipidemia	22 (28.2)	34 (44.2)	0.039^b^
Coronary heart disease	8 (10.3)	17 (22.1)	0.045^b^
Smoking	12 (15.4)	15 (19.5)	0.501^b^
MPI	0.19 [0.06–0.25]	0.25 [0.13–0.38]	0.015^a^
Baseline NIHSS	13 [6–17]	12 [6–16]	0.542^a^
Anterior circulation territory	55 (70.5)	51 (66.2)	0.567^b^
Endovascular treatment	46 (59.0)	44 (57.1)	0.817^b^
Stress hyperglycemia ratio	1.04 [0.93–1.12]	1.36 [1.30–1.54]	< 0.001^a^
Poor 3-month outcome	39 (50.0)	55 (71.4)	0.006^b^

Data are presented as median [IQR] for continuous variables, and n (%) for categorical variables.

^a^Mann-Whitney test.

^b^Chi-squared test.

Low and high SHR groups defined for SHR values < and ≥ the median SHR value in the study cohort.

Calculation of different domains of multidimensional prognostic index.

The Multidimensional prognostic index was assessed as previously reported (10). Premorbid functional status was evaluated by the activities of daily living (ADL) score (22) and by the instrumental activities of daily living (IADL) score (23) prior stroke. Comorbidity was examined using the cumulative illness rating scale [CIRS; (24)], while nutritional status was explored with the mini nutritional assessment [MNA; (25)]. Cognitive status prior to the onset of stroke was categorized as normal (0), reported cognitive impairment with no impact on ADL (0.5) and dementia (1). The Exton–Smith scale (ESS) was used to evaluate the risk of developing pressure sores (26).

The number of drugs used by patients prior stroke and the Cohabitation status, i.e., living with family (with spouse and/or other relatives and/or a caregiver were also recorded. For each domain, a tripartite hierarchy was used, i.e., 0 = no problems, 0.5 = minor problems, and 1 = major problems, the detailed rating for each domain is available as Supplementary material. The sum of the calculated scores from the eight domains was divided by eight to obtain a final MPI risk score between 0 = no risk and 1 = higher risk of mortality.

**Table 3:** Associations of stress hyperglycemia ratio and poor 3-month outcome in the study cohort.

**Independent variable**	^*^Adjusted OR (95% CI)	***p*-value**
Age	1.07 (0.99–1.15)	0.054
Baseline NIHSS score	1.16 (1.07–1.27)	< 0.001
Endovascular treatment	0.84 (0.31–2.24)	0.721
MPI	80.09 (3.90–1,644.32)	0.004
SHR	8.10 (1.68–39.09)	0.009

Poor outcome was defined as mRS ≥ 3. ORs for every 1-point increases in age, baseline NIHSS score, MPI and SHR are obtained with logistic regression analysis.

^*^Adjustment by age, baseline NIHSS score, endovascular treatment, MPI, and SHR.

CI, confidence interval; MPI, multidimensional prognostic index; mRS, modified Rankin Scale; NIHSS, National Institute of Health Stroke Scale; OR, odds ratio; SHR, stress hyperglycemia ratio.

**Table 4:** Associations of stress hyperglycemia ratio and poor 3-month outcome in robust population.

**Independent variable**	^*^Adjusted OR (95% CI)	***p*-value**
Age	1.09 (1.01–1.17)	0.022
Baseline NIHSS score	1.13 (1.03–1.24)	0.008
Endovascular treatment	1.12 (0.38–3.26)	0.839
SHR	8.62 (1.61–46.12)	0.012

Poor outcome was defined as mRS ≥ 3. Patients were classified as robust for MPI values < 0.34.

ORs for every 1-point increases in age, baseline NIHSS score, and SHR are obtained with logistic regression analysis.

^*^Adjustment by age, baseline NIHSS score, endovascular treatment, and SHR.

CI, confidence interval; MPI, multidimensional prognostic index; mRS, modified Rankin Scale; NIHSS, National Institute of Health Stroke Scale; OR, odds ratio; SHR, stress hyperglycemia ratio.

**Table 5:** Associations of stress hyperglycemia ratio and poor 3-month outcome in frail population.

**Independent variable**	^*^Adjusted OR (95% CI)	***p*-value**
Age	1.10 (0.90–1.33)	0.356
Baseline NIHSS score	1.29 (1.03–1.63)	0.028
Endovascular treatment	0.18 (0.01–2.44)	0.198
SHR	12.96 (0.14–1,231.41)	0.270

Poor outcome was defined as mRS ≥ 3. Patients were classified as frail for MPI values ≥ 0.34.

ORs for every 1-point increases in age, baseline NIHSS score, and SHR are obtained with logistic regression analysis.

^*^Adjustment by age, baseline NIHSS score, endovascular treatment, and SHR.

CI, confidence interval; MPI, multidimensional prognostic index; mRS, modified Rankin Scale; NIHSS, National Institute of Health Stroke Scale; OR, odds ratio; SHR, stress hyperglycemia ratio.

## Discussion

The present study evaluated the combined predictive value of frailty and SHR assessment for short-term outcomes in elderly patients with acute ischemic stroke.

There are few studies deeply investigating the prognostic role of SHR in acute ischemic stroke patients and they were performed mainly in Asian populations. In the meta-analysis of Huang et al. (9) on 183,588 patients, a significant increase in the incidence of poor outcome (mRS ≥ 3), mortality, neurological deficit, hemorrhagic transformation (HT), and infectious complications (pneumonia and urinary tract infection) was demonstrated in stroke patients with higher SHR, independently from the recanalization rate in patients who underwent EVT. There is no information about the potential concurrent effect of frailty in these studies. A similar effect was demonstrated in a younger population (5), where frailty cannot be considered as a confounder.

In a recent meta-analysis (11) about the prognostic role of frailty in stroke patients, including more than 20,000 subjects, the prognostic role of frailty in predicting poor outcome has been stated, but the definition of frailty and its measurement and scales showed a greater heterogeneity among individual studies, preventing further considerations. The potential association of frailty in SHR for predicting poor outcome has not been fully addressed until now and it might be a simple and useful tool using standardized scales for stratifying the patients' outcomes in an early phase and address the most critical patients from the outcome's perspective in a more individualized way.

The findings largely confirmed the predictive value of MPI (1, 12), whereas the SHR appeared to have an impact on disability in robust patients only. The study assessed frailty and stroke-specific predictors of disability in a large cohort of elderly patients treated for AIS who underwent IV endovascular treatment. Of them, about a third were classified as frail according to the MPI classification. Multidimensional frailty is a condition which has been associated with higher risk of death and worse outcomes in several acute and chronic conditions beyond the cumulative effect of age and comorbidities (10, 12–14). Our data confirm, again, that frailty is associated with short outcomes, independently from SHR, which was not associated with frailty status in the cohort. Of note, SHR emerged as important predictor of outcome in the group of robust patients only.

This raises the question whether the different sample size of groups might explain alone the findings or whether this parameter did not indeed play a role in subjects with frailer premorbid conditions with reduced brain resilience. This point might appear as contradictory, but several considerations might help to explain this lack of association. First, frailty index (as MPI) was largely applied in older patients, but SHR is not necessarily limited in its predictive value to this age range and it may interplay with individual features and even with neuroimaging markers of frailty (15), not considered in our cohort. In previous studies on EVT-treated patients with AIS, SHR was associated with early neurological deterioration and decreased likelihood of favourable outcomes (16).

In terms of age, while the conceptual underpinning of frailty extends beyond the elderly demographic, individuals aged over 65 are notably more frequently identified as frail compared to those under 65. Indeed, research indicates a progressive increase in frailty with advancing age: 4% among individuals aged 65–69 years, 7% among those aged 70–74 years, 9% among those aged 75–79 years, 16% among those aged 80–84 years, and 26% among those aged over 85 years (13). Consequently, this trend underscores why investigations into the prognostic implications of frailty in vascular diseases predominantly focus on patients aged over 65. Furthermore, advancing age is closely linked with a heightened risk of stroke, potentially influencing both the speed of stroke recovery (14) and prognosis following ischemic stroke (17). Although stress hyperglycemia ratio (SHR) is not inherently age-dependent, prior research consistently involves individuals aged over 65; for example, Dai et al. (16) reported a median (IQR) age of 70 (63–77) years in their study cohort. A very important result emerging from the study, in line with previous data (1), is the impact of both MPI and SHR independently from the role of age itself.

Frail persons are more vulnerable to a sudden change in health status following even a minor illness and stroke might be considered a major event in the natural history of these patients. Premorbid MPI is one of the several frailty measures proposed in the literature and it appears to be one of the simplest and easily applicable, allowing to compare patients according to the score. In our cohort, MPI performed well and confirmed to be a sensitive marker for predicting short- and long-term outcomes in elderly patients with AIS undergoing reperfusion therapies. It still appears premature, based on the available data, to define whether this measure can be used as a selection tool for access to treatment in management settings with reduced availability of resources. Many elements that have been documented to predict poor recovery after AIS treated with IVT and EVT, even with prompt recanalization of the occluded vessel, were not taken into consideration in this cohort, favouring a tool such as MPI, easily usable in the absence of diagnostic techniques advanced. One of these is, for example, the presence of moderate or severe leukoaraiosis in baseline neuroimaging investigations, being a marker of small vessel disease and reduced cognitive reserve (18, 19). A further finding is that in the subgroup of “robust” patients according to MPI, the SHR on admission was significantly associated with the prognosis together with baseline NIHSS and age. Because of the small number of included patients, we did not analyse whether the significance of SHR was different between diabetic and non-diabetic patients. The significance of the SHR only in the subgroup of robust patients makes it a different index compared to frailty measures. It could be hypothesized to use it in non-frail patients not only as an outcome indicator but also as a tool to identify a subset of patients at a greater risk to be subjected to closer control of metabolic parameters.

It seems that, when present, frailty is a strong predictor of a worse outcome at 3 months. Conversely, in not frail patients, metabolic status, measured by SHR, could help to identify a subset of more vulnerable AIS patients. This is coherent with the non-linear dose-response relationship demonstrated on larger samples (20) between SHR and poor outcome in AIS patients. Moreover, the need of further studies to detail this association is supported from a recent demonstration that the neutrophil counts and neutrophil-to-lymphocyte ratio (NLR) are positively associated with SHR levels in AIS patients (21).

However, this hypothesis needs to be confirmed in larger on-going longitudinal cohorts stratified for etiological subtype of stroke, treatment management, neuroimaging and systemic inflammatory/neuronal and glial markers.

### Main findings

MPI may appear to be a more relevant marker than the SHR in term of functional outcome in geriatric patients. MPI incorporates more items of morbidity and disability such as number and severity of comorbidities, functional, cognitive, nutritional, and social status, that altogether influence more concretely patients' functional recovery.

### Limitations of the study and conclusion

Limitations of this study include its retrospective design, the relatively small sample size, underpowered for subgroup analyses, and the lack of detailed neuroimaging data.

Moreover, our data cannot be extended to patients who did not receive thrombolytic therapy or underwent thrombectomy.

Further longitudinal multicentre and prospective studies are needed to confirm our results and to validate the differential role of MPI and SHR in AIS patients. In conclusion, MPI is associated with the 3-months outcome in our cohort, in particular with good outcome. Conversely, SHR seems to be associated with a 3-months poor outcome in “robust” patients but not in frail patients.

## Data Availability

The raw data supporting the conclusions of this article will be made available by the authors, without undue reservation.
