# Supplementary material for: Premorbid frailty, stress hyperglycemia ratio, and functional outcome in patients with acute ischemic stroke
Source: Front Neurol. 2024 Oct 24;15:1463814. doi: 10.3389/fneur.2024.1463814 (PMC11540662; doi:10.3389/fneur.2024.1463814)
Supplement: Supplementary file 1 [file Table_1.docx]

Supplementary Table 1. Baseline characteristics of study participants according to the study center

|  | **Center 1 (n=95)** | **Center 2 (n=60)** | **sssp value** |
| --- | --- | --- | --- |
| **Age,** yr  **Diabetes mellitus**  **Hypertension**  **Atrial fibrillation**  **Dyslipidemia**  **Coronary heart disease**  **Smoking**  **MPI**  **Baseline NIHSS**  **Anterior circulation territory**  **Endovascular treatment**  **Stress hyperglycemia ratio**  **Poor 3-month outcome** | 77 [72-81]  35 (36.8)  80 (84.2)  28 (29.5)  31 (32.6)  16 (16.8)  15 (15.8)  0.19 [0.13-0.31]  8 [4-13]  53 (55.8)  30 (31.6)  1.20 [1.04-1.35]  53 (55.8) | 77 [71-82]  12 (20.0)  42 (70.0)  22 (36.7)  25 (41.7)  9 (15.0)  12 (20.0)  0.22 [0.13-0.35]  16 [14-18]  53 (88.3)  60 (100.0)  1.20 [1.04-1.41]  41 (68.3) | 0.580^a^  0.026^b^  0.035^b^  0.351^b^  0.254^b^  0.761^b^  0.501^b^  0.845^a^  <0.001^a^  <0.001^b^  <0.001^b^  0.772^a^  0.119^b^ |

Data are presented as median [IQR] for continuous variables, and n (%) for categorical variables. ^a^Mann-Whitney test. ^b^Chi-squared test.
